# Biological Control of Severe Fungal Phytopathogens by *Streptomyces albidoflavus* Strain CARA17 and Its Bioactive Crude Extracts on Lettuce Plants

**DOI:** 10.3390/plants12102025

**Published:** 2023-05-18

**Authors:** Antonia Carlucci, Andrea Sorbo, Donato Colucci, Maria Luisa Raimondo

**Affiliations:** Department of Agricultural Sciences, Food, Natural Resources and Engineering, University of Foggia, Via Napoli 25, 71122 Foggia, Italy; andreasorbo92@gmail.com (A.S.); donatocolucci.dc@gmail.com (D.C.); marialuisa.raimondo@unifg.it (M.L.R.)

**Keywords:** microbial antagonist, *Athelia rolfsii*, *Sclerotinia sclerotiorum*, biological control, crude extracts

## Abstract

Lettuce crop is an important horticultural crop of several Mediterranean countries, including Italy. The Italian region which is a major producer of lettuce crops is Apulia, where this crop is cultivated in open fields an in greenhouses. Since several microbial pathogens are responsible for important diseases found on lettuce produced in greenhouses, in this study, the experimental activities focused on the most severe fungal soilborne pathogens, i.e., *Sclerotinia sclerotiorum* and *Athelia rolfsii*. Their control is often performed with fungicides which cause public concern over the environment and human health. The main aims of this study were to determine the biocontrol efficacy of a *Streptomyces* strain in vitro and in vivo conditions on lettuce seedlings against *Athelia rolfsii* and *Sclerotinia sclerotiorum* as severe fungal soilborne pathogens through the application of its vegetative propagules and putative bioactive crude extracts via filtrate culture. The results obtained confirm a significant effectiveness of CARA17 strain to control the severity of both fungal soilborne pathogens during two different experiments: when it is used as vegetative propagules and as a culture filtrate containing putative bioactive metabolites in vitro and in vivo conditions. These preliminary results demonstrated that the actinomycetes CARA17 strain is valid as a biocontrol agent (BCA) against both the severe phytopathogens used in this study. The biocontrol action performed from the CARA17 strain is clearly and mainly due to the putative bioactive crude extracts produced, but further studies are necessary to identify which metabolites (polyphenols, terpenes, fatty acids, etc.) are produced from this *Streptomyces* strain.

## 1. Introduction

Lettuce (*Lactuca sativa* L.) is a common horticultural plant for countries within the Mediterranean basin, and it is one of the most economically important vegetable crops worldwide. In particular, this crop is of great economic importance in Italy, as it is the third most-consumed product after potatoes (700 thousand tons) and tomatoes (580 thousand tons). In Italy, lettuce is represented by several varieties and is produced in both greenhouse and open-field conditions, at 480 thousand tons. Apulia is the country’s major lettuce-producing region with 609 thousand hectares; yet, lettuce is produced in all Italian regions [1]. Lettuce plants are very susceptible to several microbial pathogens, including fungal ones. In particular, *Bremia lactucae*, responsible for downy mildew, and *Botrytis cinerea*, responsible for grey mold, are two of the most destructive aerial pathogens of lettuce plants worldwide [2]. Other important phytopathogens are those that are soilborne, causing general symptoms such as: basal rot disease, which is associated with several species of *Pythium* and *Phytophthora cryptogea*; basal leaf decay, which is associated with *Rhizoctonia solani*, *Sclerotinia sclerotiorum,* and *S. minor* [3,4,5,6,7]; southern blight disease, caused by *Athelia rolfsii*; and vascular diseases (wilt), associated mainly with *Fusarium oxysporum* f.sp. *lactucae* [8]. All of the abovementioned phytopathogens are very aggressive to lettuce plants, but the most destructive are *Sclerotinia* spp. and *Athelia rolfsii*, which result in very difficult to control problems in greenhouse and open-filled crops, leading to significant crop yield losses [6,9].

*Sclerotinia* spp. and *A. rolfsii* are soilborne necrotrophic fungal plant pathogens that are considered the most devastating of various plant species, are able to cause lettuce drop, stem and pod rots, and southern blight and damping-off diseases. These fungi have been found to be very severe, especially when they infect seedlings, causing high economic losses. The severity of both *Sclerotinia* spp. and *A. rolfsii* is due to an abundant production of hyphae and sclerotia as asexual structures that allow the pathogens to survive in soil in the absence of the host plants for very long periods. For these reasons, the control of plant diseases has become a serious challenge for farmers [10]. To control these phytopathogens, fungicide are often used during conventional and/or integrated management approaches, which lead to harmful effects and chemical residues for plants, humans, and the environment. Alternative control means with reduced or without damages for the environment and human health are based on antagonistic micro-organism application [10].

Therefore, a substantial amount of research has regarded biological control as a promising alternative approach to controlling soilborne diseases in sustainable and organic agriculture. Indeed, many microbial antagonists have been demonstrated to be able to control a large number of pathogens, such as fungi and bacteria. For instance, *Trichoderma* spp. as biocontrol agents (BCAs) are the most important and are more widely used against different fungal pathogens such as *Fusarium oxysporum* f. sp. *lactucae* [11], *Sclerotinia sclerotiorum,* and *Chalara thielavioides* [12]. *Pseudomonas* spp. is used against *Verticillium dahliae* [13], and *Bacillus* spp. against several phytopathogens [14]. It is known that actinomycetes are extensively present micro-organisms in the environment that have potential to produce bioactive compounds against phytopathogens [15,16]. Among the actinomycetes, *Streptomyces* is the largest genus belonging to the family Streptomycetaceae [17], with interesting potential to produce compounds for antibiotics and agro-antibiotics, or other bioactive compounds [18,19,20]. In particular, *Streptomyces* spp. are the most spread Gram-positive filamentous bacteria that are ubiquitous in the soil as free-living organisms and symbionts of plants and animals [14]. They are known for producing a wide variety of active biological compounds, are used in agriculture as plant growth promoters, and are known to be effective as BCAs against a large number of plant pathogens [14].

Kurnianto et al. [21] demonstrated that *Streptomyces* isolates were able to produce antibacterial metabolites crude extracts effective to inhibit the growth of some bacteria such as *Staphylococcus aureus*, *Bacillus cereus*, and *Pseudomonas aeruginosa*. Although a few studies have been carried out to assess the biocontrol efficacy and mechanism of *Streptomyces* spp. [15,17], to date, there has been no research related to the control of soilborne pathogens on lettuce crops.

Therefore, the main aims of the present study were: (i) to assess the effectiveness of *Streptomyces* strain in dual culture and its bioactive crude extracts against *Athelia rolfsii* and *Sclerotinia sclerotiorum* in vitro conditions; (ii) to determine the biocontrol efficacy of a *Streptomyces* strain on lettuce seedlings against both severe fungal soilborne pathogens in vivo conditions, by application of its propagules; and (iii) to evaluate the antagonistic efficacy of putative metabolites (bioactive compounds) produced from *Streptomyces* strain as metabolite crude extracts in vivo conditions.

## 2. Results

### 2.1. Assessment of Inhibitory Activity (IA) by CARA17 Strain in Dual Culture and As Culture Filtrate Added to Culture Medium

The identification and characterization of fungal phytopathogens and CARA17 strain, as well as the antagonistic activity in dual culture were ascertained as reported in previous research [15]. Regarding the antagonistic activity played by the culture filtrate of the CARA17 strain, the results are reported in Table 1. According to Shapiro–Wilk tests, the data followed normal distributions 0.75 (*p* < 0.05), and according to Levene tests, the homogeneity of variance was also significant (F = 4.02; *p* < 0.05). One-way ANOVA demonstrated that significant differences in inhibitory activity (IA) were recorded when the culture filtrates of CARA17 strain were pretreated at two temperatures (121 and 100 °C) and added to a GYMA medium with different amounts (15, 30 and 50%; *v/v*) against the fungal pathogens *A. rolfsii* and *S. sclerotiorum*. Indeed, Table 1 shows that all temperatures used in order to inactivate the vegetative propagules of the actinomycete contained in culture filtrates and the amounts of culture filtrates used were able to inhibit the growth of soilborne fungi tested. The most significant IA percentages recorded were those correlated with lower temperatures (100 °C) and with major amounts (50%) of culture filtrates added to the GYMA medium (Table 1).

**Figure 1:**
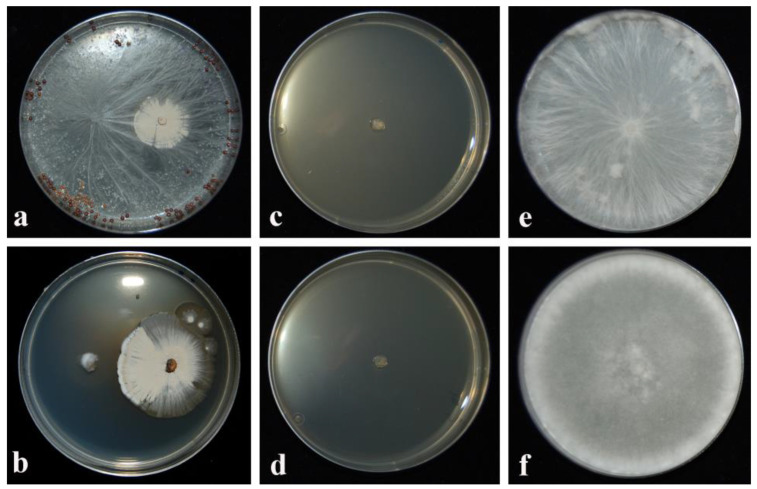
Inhibitory activity played by CARA17 strain: in dual culture with *Athelia rolfsii* (**a**) and *Sclerotinia sclerotiorum* (**b**) after 21 days; GYMA medium amended with 50% (*v:v*) of its culture filtrate pretreated at 100 °C against *A. rolfsii* (**c**) and *S. sclerotiorum* (**d**). *A. rolfsii* (**e**) and *S. sclerotiorum* (**f**) on GYMA medium not amended after 6 days.

The IAs played by the CARA17 strain in dual cultures with fungal pathogens and by its culture filtrate added into the GYMA medium are reported in Table 2. One-way ANOVA demonstrated that significant differences in inhibitory activity (IA) by the CARA17 strain against fungal pathogens were observed. In particular, in dual culture, *S. sclerotiorum* resulted to be sensible (IA, 100%) regarding to the presence of CARA17 strain, while *A. rolfsii* showed no inhibition (IA, 0.0%) after 21 days (Figure 1). On a GYMA medium amended with 50% of the culture filtrate of CARA17 strain pretreated at 100 °C, both of the fungal phytopathogens resulted in an inhibition at 100% after 6 days (Table 2; Figure 1).

### 2.2. Assessment of Antagonistic Effectiveness of CARA17 Strain on Lactuca sativa L. Seedlings against Fungal Soilborne Pathogens In Vivo

The results obtained from in vivo experiments carried out in greenhouses on lettuce seedlings are reported in Table 3. The symptoms observed on the roots mainly consisted of rotting, while on the leaves, they consisted of marginal necrosis and browning, and light necrosis. The treatment by dipping with propagule inoculum of CARA17 protected the 98.3 and 88.3% of lettuce plants (healthy seedlings) against *S. sclerotiorum* and *A. rolfsii*, respectively, while the treatment by dipping in the filtrate inoculum of CARA17 resulted in a less efficacious protection of 86.7 and 80.0% of the lettuce plants, respectively.

When the soilborne pathogens were used without CARA17 inoculum, all inoculated lettuce seedlings resulted symptomatic. No symptoms were observed on all seedlings (100%) treated with both propagule and filtrate inocula of CARA17 and with tap water. The disease severity (DS) values assessed by observations carried out on the roots of lettuce seedlings treated by dipping in CARA17 propagule inoculum were 0.6 for *S. sclerotiorum* and 1.2 for *A. rolfsii*. Similar DS values (0.6 and 1.6) were collected when the lettuce seedlings were treated by dipping in CARA17 filtrate inoculum by *S. sclerotiorum* and *A. rolfsii*, respectively. However, the DS values collected from the roots of seedlings treated only with fungal soilborne pathogens were 3.9 for *S. sclerotiorum* and 5.0 for *A. rolfsii*. No DS values were recorded from lettuce seedlings used as control treated with both propagule and filtrate inocula of CARA17 and with the tap water. The DS values followed a similar trend regarding the symptoms observed on leaves. The percentages of the re-isolation of both fungal pathogens from symptomatic seedlings were higher than from healthy seedlings (Table 3).

## 3. Discussion

It is known that the control of several severe fungal phytopathogens is very difficult, and increasing amounts of fungicides are necessary despite consumer demands for products with low or no chemical residues for the health of both humans and ecosystems. To satisfy consumers, many studies have been conducted with the aim to detect and prove the potential ability of biological control agents (BCAs) to control fungal and bacterial phytopathogens [21,22,23,24,25,26]. The *Streptomyces* species is known as an effective biocontrol agent against numerous plant pathogens [15,22,23,24,25] because it has shown to be able to produce bioactive compounds [21,26,27] as well as to reduce or inhibit the mycelial growth of several fungal pathogens [14,25,27,28]. Many *Streptomyces* strains used as BCAs have shown various degrees of disease-suppressive activity, depending on whether they were tested in laboratories, fields, or greenhouses and the methods used [25,26,27]. Newitt et al. [29] reported different studies related to *Streptomyces* spp. to control several diseases occurring on cereal crops carried out in different conditions (laboratory, greenhouse and open-field). Moreover, Law et al. [30] described that different *Streptomyces* spp. are able to control *Magnaporthe oryzae* as a causal agent of rice blast; Kaur et al. [22] and Colombo et al. [31] showed the ability of a *Streptomyces* spp. to control *Fusarium moniliforme* and *F. graminearum*. Similar results were obtained by Carlucci et al. [15] that discussed the ability of *Streptomyces albidoflavus* CARA17 strain to control several severe fungal phytopathogens occurring on fennel plants in southern Italy in vitro and in vivo conditions, such as *Athelia rolfsii*, *Fusarium oxysporum*, *Plectosphaerella ramiseptata*, *Sclerotina sclerotiorum,* and *Verticillium dahliae*.

The same actinomyces strain has been used in the current study in order to demonstrate that it is able to control *Sclerotina sclerotiorum* and *Athelia rolfsii* on lettuce plants. In particular, two different methods of inoculation were tested in this study in order to verify the ability of crude extracts from a *Streptomyces* CARA17 strain at inhibiting the abovementioned soilborne pathogens in vitro and in vivo conditions.

The culture filtrate of a CARA17 strain was thermally pretreated at two different temperatures to inactivate vegetative propagules of BCA *Streptomyces* before adding to a culture medium at three different concentrations (15, 30, and 50%) during the assessment of essays in vitro conditions. In this case, the culture filtrate was able to present significant inhibitory activity percentages against the mycelial growth of *S. sclerotiorum* and *A. rolfsii* at already lower concentrations when the culture filtrate was thermally pretreated at 100 °C. These results highlight that the higher concentrations of culture filtrates are more efficacious, and the high temperatures of pretreatment can reduce the effectiveness of putative bioactive compounds because, most likely, these are thermally sensitive. For these reasons, for setting up the greenhouse experiment, the culture filtrate of CARA17 strain was thermally treated at 100 °C. The results obtained from the greenhouse experiment demonstrated that the culture filtrate was able to significantly prevent the severity of fungal pathogens used, although the inoculations carried out with propagule inoculum showed a better prevention on the lettuce seedlings with the higher number of healthy seedlings. The better effectiveness of the propagule inoculum than filtrate inoculum is probably due to synergic actions played from crude extracts and vegetative propagules according according to Passari et al.’s work [32], which highlighted an enhanced systemic resistance induced, as well as De-Olivera et al. [33], whose work highlighted instead an enhanced the hyperparasitism mechanism. Moreover, this study highlighted that the *Streptomyces albidoflavus* CARA17 strain was more efficacious in protecting the lettuce seedlings against *S. sclerotiorum* than *A. rolfsii* in both inoculation kinds of the antagonist. These data confirmed the higher severity of *A. rolfsii*.

Based on the results obtained from this study, we suggest that the CARA17 strain might be considered a suitable biological control agent (BCA), similarly to other antagonistic fungal and bacterial micro-organisms, as well as *Conyothirium minitans* [34], *Trichoderma* spp. [35,36], *Pseudomonas brassicacearum* [37,38], and *Bacillus amyloliquefaciens* [38].

The current results discussed here confirm the previous data [15] and encourage further studies to detect and characterize the chemical nature of crude extracts released from CARA17 strain in liquid cultures, and then to assay the secondary metabolites directly onto horticultural seedlings or seeds in order to protect them before and/or after transplantations in open fields. Further studies are needed to test their putative toxicity against plants and final consumers as well as their persistence in edible products.

## 4. Materials and Methods

### 4.1. Actinomycetes Strain and Soilborne Phytopathogens

The CARA17 strain of *Streptomyces albidoflavus* used in the research activities described herein was isolated and characterized in early research as reported by Carlucci et al. [15]. The phytopathogens *Athelia rolfsii* and *Sclerotinia sclerotiorum* used in this study were isolated from fennel plants during previous surveys carried out in the northern Apulia region (2017–2020). The assessment of antagonistic activity played from the Streptomyces strain CARA17 in vitro conditions against *A. rolfsii* and *S. sclerotiprum* was performed as described by Carlucci et al. [15]. All fungal strains used here were maintained in the laboratory of the Plant Pathology and Diagnosis of Department of Sciences Agriculture, Food, Natural resources and Engineering (DAFNE) at the University of Foggia, Italy.

### 4.2. Assessment of Inhibitory Activity by CARA17 Strain in Dual Culture and As Culture Filtrate

To assess the inhibitory activity of CARA17 strain against *Sclerotinia sclerotiorum* and *Athelia rolfsii*, two kinds of experiments were performed. The first consisted of preparation of dual culture as described by Carlucci et al. [15]. The second consisted of preparation of culture filtrate of CARA17 strain by inoculation of a broth medium. In particular, the broth medium used was GYM (broth of GYM; glucose 4 g, yeast extract 4 g, malt extract 10 g, distilled water 1000 mL; adjusting the pH at 7.2 by NaOH 3 M) inoculated with 1 × 10^6^ cfu per ml of CARA17 propagules collected from 21 day-old colony, incubated at 28 ± 3 °C at 75 rpm for 21 days, and then filtered on Whatman filter, 5.5 µm in size. The filtered culture was evaluated for antifungal activity by inclusion of 15, 30, and 50% (*v:v*) in GYMA medium and early-sterilized at 115 °C for 10 min. After adding CARA17 culture filtrate, the GYMA (GYM; 15 g Agar Difco, Sigma-Aldrich, Milan, Italy) was again sterilized at 100 and 121 °C for 10 min. The Petri plates obtained were inoculated with an agar-mycelium disc (5 mm diameter) from both phytopathogens fungi (*A. rolfsii* and *S. sclerotiorum*), and incubated at 28 ± 3 °C for six days to evaluate the inhibition activity played from CARA17 filtrate. Five replicates were performed for both soilborne fungal pathogens. Plates inoculated with phytopathogens and the sterile agar discs were used as controls. The inhibitory activity (IA) was calculated as the percentage of mycelium growth inhibition compared to the control according to Carlucci et al. [15]. Before performing statistical analysis, the percentage data of inhibition activity were arcsine-root-square transformed according to Carlucci et al. [15]. One-way ANOVA analysis was performed using Statistica, version 6 (StatSoft, Hamburg, Germany) to assess the significant differences of inhibition activity values. Fisher’s test was used as a post hoc test (*p* = 0.01).

### 4.3. Assessment of Antagonistic Effectiveness of CARA17 Strain on Lactuca sativa L. Seedlings against Fungal Soilborne Pathogens In Vivo

Inoculation preparation of biological control agent CARA17 strain: Two different inoculum kinds of CARA17 strain were prepared. One consisted of preparing the inoculum solution with the CARA17 strain propagules by collecting spores and small fragments of mycelium, scraped from surface the of 21 day-old Streptomyces colonies, grown on PDA (potato dextrose agar, Oxoid; Sigma-Aldrich) medium at 28 ± 3 °C in darkness until reaching a concentration of 1 × 10^7^ cfu/mL in sterile Tween 20 solution (0.2%). The other inoculum kind consisted of preparing a liquid culture of CARA17 strain grown into liquid medium (broth of GYM) at 28 ± 3 °C in darkness at 75 rpm. After 21 days, the antagonist culture was filtered on Whatman paper filters (size 5.5 µm) and subjected to 100 °C for 10 min to inactivate all vegetative propagules of antagonist.

Inoculation preparation of soilborne pathogens, *A. rolfsii* and *S. sclerotiorum*: The inoculum solution with each fungal pathogen was prepared by scraping from the surface of 21 day-old colonies grown on PDA medium at 21 ± 3 °C in darkness until reaching a concentration of 1 × 10^7^ cfu/mL in sterile Tween 20 solution (0.2%).

The experimental design was performed as two independent batches at the end of August 2022 and consisted of two different inoculum kinds of CARA17 strain as described above, against two fungi targets such as *A. rolfsii* and *S. sclerotiorum* as severe fungal pathogens of *Lactuca sativa* var. *Iceberg* using 30 day-old seedlings.

The experiment consisted of setting up of two main trials: (a) dipping twelve lettuce seedlings in the inoculum solution of propagules of CARA17 strain for 12 h (propagule inoculum); (b) dipping twelve lettuce seedlings in the CARA17 strain inoculum solution of culture filtrated for 12 h (filtrate inoculum). After dipping, the lettuce seedlings were transplanted in pots containing 1.5 kg of soil and peat (3:1) (sterilized early twice at 121 °C for 30 min and kept for 20 days in a controlled chamber at 25 ± 3 °C, in 70% relative humidity, and under natural light) and wetting them with 500 mL of irrigation water. Subsequently, 50 mL of inoculum solution of both fungal soilborne pathogens were poured into the soil of each pot around the collar of the lettuce seedlings.

As control trials, pots containing lettuce seedlings treated with both kinds of CARA17 strain inocula (propagule and filtrate inocula), two other trials treated only with each fungal pathogen (no treatment), and another trial not treated with either the CARA17 strain or the fungal soilborne pathogens (control, tap water) were prepared. Each trial was replicated five times. The pots with lettuce seedlings were placed in a greenhouse with temperature and humidity not controlled. During the growth of the seedlings, no fertilizers, pesticides or fungicides were used. They were only subjected to irrigation practice when necessary, using the same water volumes for each pot. After 100 days, the lettuce plants were gently removed from the pots, the roots and collars were carefully washed, and the presence/absence of browning/rot symptoms observed on the roots and leaves were evaluated and described using an empirical scale from 0 to 5, where 0 = no symptoms observed; 1 = 1–20%; 2 = 21–40%; 3 = 41–60%; 4 = 61–80%; and 5 = 81–100%. The disease severities (DS) on the roots and leaves were determined according to the following formula:

$$\mathrm{DS}=\frac{\sum_{}\left(Number\;of\;observ\times values\;score\right)}{Total\;number\;of\;cases}$$

All fungi underwent re-isolation from the root of the inoculated plants to fulfil Koch’s postulates.

## Figures and Tables

**Table 1:** Assessment of the mycelium growth of fungal soilborne pathogens on GYMA medium with different amounts of added CARA17 strain culture filtrates thermally pretreated at different temperatures.

	CARA17 Strain—Culture Filtrate			
Soilborne Fungus	Thermally Pretreated	Quantity Added to Culture Medium (%) (*v/v*)	Inhibitory Activity (%)	
			Mean	SD
*Athelia rolfsii*	121 °C	15	22.81 A *	4.94
*Sclerotinia sclerotiorum*	121 °C	15	50.50 B	13.50
*Sclerotinia sclerotiorum*	121 °C	30	85.68 C	1.18
*Sclerotinia sclerotiorum*	121 °C	50	85.94 C	0.28
*Athelia rolfsii*	100 °C	15	88.99 C	1.24
*Athelia rolfsii*	121 °C	30	89.56 C	1.81
*Athelia rolfsii*	121 °C	50	90.59 CD	1.15
*Sclerotinia sclerotiorum*	100 °C	15	91.09 CD	5.63
*Athelia rolfsii*	100 °C	30	93.05 CDE	6.19
*Sclerotinia sclerotiorum*	100 °C	30	96.92 DE	0.44
*Sclerotinia sclerotiorum*	100 °C	50	100.00 E	0.00
*Athelia rolfsii*	100 °C	50	100.00 E	0.00

* Values followed by a different capital letter in columns are significantly different according to Fisher’s test (*p* < 0.01).

**Table 2:** Inhibitory activity percentage (IA) by CARA17 strain against mycelial growth of soilborne pathogens in vitro (dual culture).

	Inhibitory Activity (IA) %			
Fungal Soilborne Isolates	Dual Culture after 21 Days		GYMA Amended with 50% of CARA17 Culture Filtrate after 6 Days	
	Mean	Min–Max ^b^	Mean	Min–Max
*Athelia rolfsii*	0.00 B ^a^	0.00–0.00	100.00 A	100.00–100.00
*Sclerotinia sclerotiorum*	100.00 A	100.00–100.00	100.00 A	100.00–100.00

^a^ Data followed by different capital letters within the columns are significantly different (Fisher’s tests; *p* < 0.01); ^b^ minimum and maximum values detected (five observations).

**Table 3:** Effectiveness of CARA17 strain as antagonist for controlling disease severity (DS) against fungal soilborne pathogens on lettuce seedlings.

Treatment with CARA17 Strain by Dipping for 12 h	Fungal Pathogen	Phytosanitary Status of Lettuce Seedlings		Disease Severity and Symptom Description				Pathogen Re-Isolation from Root *n*(/%) *	
		Healthy Seedlings (*n*/%)	Symptomatic Seedlings (*n*/%)	DS	Root	DS	Leaves	Healthy Seedlings	Symptomatic Seedlings
Filtrate inoculum ^a^	*A. rolfsii*	48 (80.0)	12 (20.0)	1.6	Root rot	1.0	Marginal necrosis	2 (4.2)	10 (84)
Filtrate inoculum ^a^	*S. sclerotiorum*	52 (86.7)	6 (13.3)	0.6	Root rot	0.4	Marginal browning and light necrosis	1 (1.9)	6 (100)
Propagule inoculum	*A. rolfsii*	53 (88.3)	7 (11.7)	1.2	Root rot	0.6	Marginal necrosis	4 (7.5)	7 (100)
Propagule inoculum	*S. sclerotiorum*	59 (98.3)	1 (1.7)	0.6	Root rot	0.4	Marginal browning and light necrosis	6 (10.2)	1 (100)
No treatment ^b^	*A. rolfsii*	0 (0.0)	60 (100.0)	5.0	Root rot	5.0	Damping off	0 (0.0)	58 (97)
No treatment ^b^	*S. sclerotiorum*	0 (0.0)	60 (100.0)	3.9	Root rot	4.5	Damping off	0 (0.0)	60 (100)
Filtrate inoculum	*-*	60 (100.0)	0 (0.0)	0.0	No disease symptoms	0.0	No disease symptoms	-	-
Propagule inoculum	*-*	60 (100.0)	0 (0.0)	0.0	No disease symptoms	0.0	No disease symptoms	-	-
Control, tap water ^c^	-	60 (100.0)	0 (0.09	0.0	No disease symptoms	0.0	No disease symptoms	-	-

* Data recorded from root of healthy and symptomatic seedlings. ^a^ Culture filtrate thermally pretreated (100 °C; 15 min); ^b,c^ dipping only in tap water.
